# Projected detection of active pulmonary TB through the Japan pre-entry TB screening programme

**DOI:** 10.5588/ijtldopen.26.0270

**Published:** 2026-10-12

**Authors:** A. Ohkado, S. Lee, N. Yamada

**Affiliations:** 1 Department of Epidemiology and Clinical Research, Research Institute of Tuberculosis (RIT), Japan Anti-Tuberculosis Association (JATA), Tokyo, Japan;; 2 Centre for JPETS Quality Assessment, Research Institute of Tuberculosis (RIT), Japan Anti-Tuberculosis Association (JATA), Tokyo, Japan;; 3 Centre for International Cooperation and Global TB Information, Research Institute of Tuberculosis (RIT), Japan Anti-Tuberculosis Association (JATA), Tokyo, Japan.

**Keywords:** tuberculosis, TB prevalence, foreigners, immigrants, Asia

## Abstract

**BACKGROUND:**

This study aimed to estimate the number of bacteriologically confirmed pulmonary TB (BC-PTB) cases expected to be detected and the number needed to screen (NNS) to detect one BC-PTB case by the Japan Pre-entry Tuberculosis Screening (JPETS) programme.

**METHODS:**

This descriptive study estimated the number of cases with BC-PTB to be detected and the NNS required to detect one case using the JPETS programme, based on national TB prevalence survey data from the six JPETS target Asian countries and immigration statistics in Japan.

**RESULTS:**

Among JPETS applicants, the estimated total number of BC-PTB cases diagnosed per year was 1,997 (95% confidence interval [CI]: 1,910–2,086) when adjusted for sex and 1,323 (95% CI: 1,253–1,396) when adjusted for age group. The estimated NNS to identify one BC-PTB case was 227 cases (95% CI: 217–237) when adjusted for sex and 319 cases (95% CI: 302–337) when adjusted for age.

**CONCLUSION:**

The JPETS is projected to identify approximately 1,300 BC-PTB cases per year across six Asian countries. When selecting countries for JPETS, consideration of both the number of TB cases in Japan and the TB prevalence in target countries may enhance screening effectiveness.

In 2021, Japan recorded a rate of 9.1 newly notified active TB cases per 100,000 population, dropping as low as 8.1 in 2024.^1,2^ While more than two thirds of all newly notified active TB cases were among people aged over 60 years, reflecting the ageing population in Japan, 19.7% (1,980 cases) of all cases (10,051 cases) notified in 2024 were foreign-born, a significant increase from 16.0% in 2023.^2^ This trend is expected to continue. Hence, reducing the number of foreign-born TB cases in Japan is important to further reduce the total number of cases. In February 2018, the Ministry of Health, Labour and Welfare (MHLW) introduced the Japan Pre-entry Tuberculosis Screening (JPETS) programme for six countries (the Philippines, Nepal, Vietnam, Indonesia, Myanmar, and China) that account for more than 80% of TB cases reported among foreign-born TB cases. Although the start of JPETS was postponed due to the global COVID-19 pandemic, the Government of Japan decided to commence this programme in stages by the end of fiscal year 2024.^3^ Under the coordination of the MHLW, the Ministry of Foreign Affairs, and the Immigration Services Agency, the programme was introduced, initially with three countries (the Philippines, Nepal, and Vietnam) that were ready to cooperate. In December 2024, the MHLW published the ‘Japan Pre-Entry Tuberculosis Screening Technical Instructions, December 2024 Edition’ to clarify the specific procedures for this programme at designated medical institutions (Panel Clinics) in the target countries.^4^ JPETS intends ‘to detect the suspected presence of active TB, as well as to contribute to applicants’ health and TB control in their home countries of applicants by early diagnosis and appropriate treatment of TB’.^4^ It is ‘a TB test to certify with the TB Clearance Certificate that foreign nationals who intend to enter Japan from target countries and stay for a medium- to long-term do not have active tuberculosis by undergoing a chest X-ray examination’.^5^

As this is a new programme, it is unclear to what extent JPETS can detect cases with bacteriologically confirmed active pulmonary TB (BC-PTB). This study, therefore, aimed to estimate the number of cases with BC-PTB that JPETS is expected to detect in the six target countries.

## METHODS

This was a descriptive study aiming to estimate the number of cases with BC-PTB who would be detected by the JPETS programme using national TB prevalence survey (NPS) data from the WHO and national TB control programmes in the six targeted countries.^6–10^ We used the most up-to-date available NPS data for each country available at the time of the present study. We calculated the number of JPETS applicants expected to be diagnosed with BC-PTB, adjusting for sex and age group for each country, using NPS data and Japan’s immigration statistics.^6–12^ We further calculated the number needed to screen (NNS) to detect a case with BC-PTB, defined as the number of JPETS applicants screened divided by the number of cases with BC-PTB detected. We applied 95% confidence intervals (CIs) for the number of cases with BC-PTB and the NNS, as calculated in the NPS report for each country.^6–10^ We calculated 95% CIs for the overall number of cases with BC-PTB and the NNS to obtain the approximate values assuming the Poisson distribution. The years of the NPS conducted varied across the six countries: 2016 in the Philippines, 2018–2019 in Nepal, 2017 in Vietnam, 2013–2014 in Indonesia, 2017–2018 in Myanmar, and 2010 in China.

We excluded the 0–14-year age group from the analysis because BC-PTB prevalence data for this age group were not available in the NPS. In JPETS, some people from eligible countries are exempt from obtaining a TB-free certificate to enter and stay in Japan, such as those entering under specific schemes and those holding a re-entry permit.^5^ However, we did not exclude these individuals as the total number of people exempt from this requirement in each country was not publicly available at the time this study was conducted.^5^

### Ethical statement

As only publicly available data were used, this study did not require institutional review board approval. Further, we did not use any personally identifiable data during the study in the publication process.

## RESULTS

Among the JPETS applicants in the six target countries, the estimated total number of BC-PTB cases diagnosed through TB screening per year was 1,997 (95% CI: 1,910–2,086) when adjusted for sex and 1,323 (95% CI: 1,253–1,396) when adjusted for age group, as presented in Figure 1. The estimated number of male applicants diagnosed with BC-PTB was more than three times that of female applicants (1,504 male cases vs. 493 female cases). The estimated number of applicants in the 25–34-year-old age group (555 applicants, 95% CI: 510–603) was the largest among all age groups (Figure 1).

**Figure 1.**
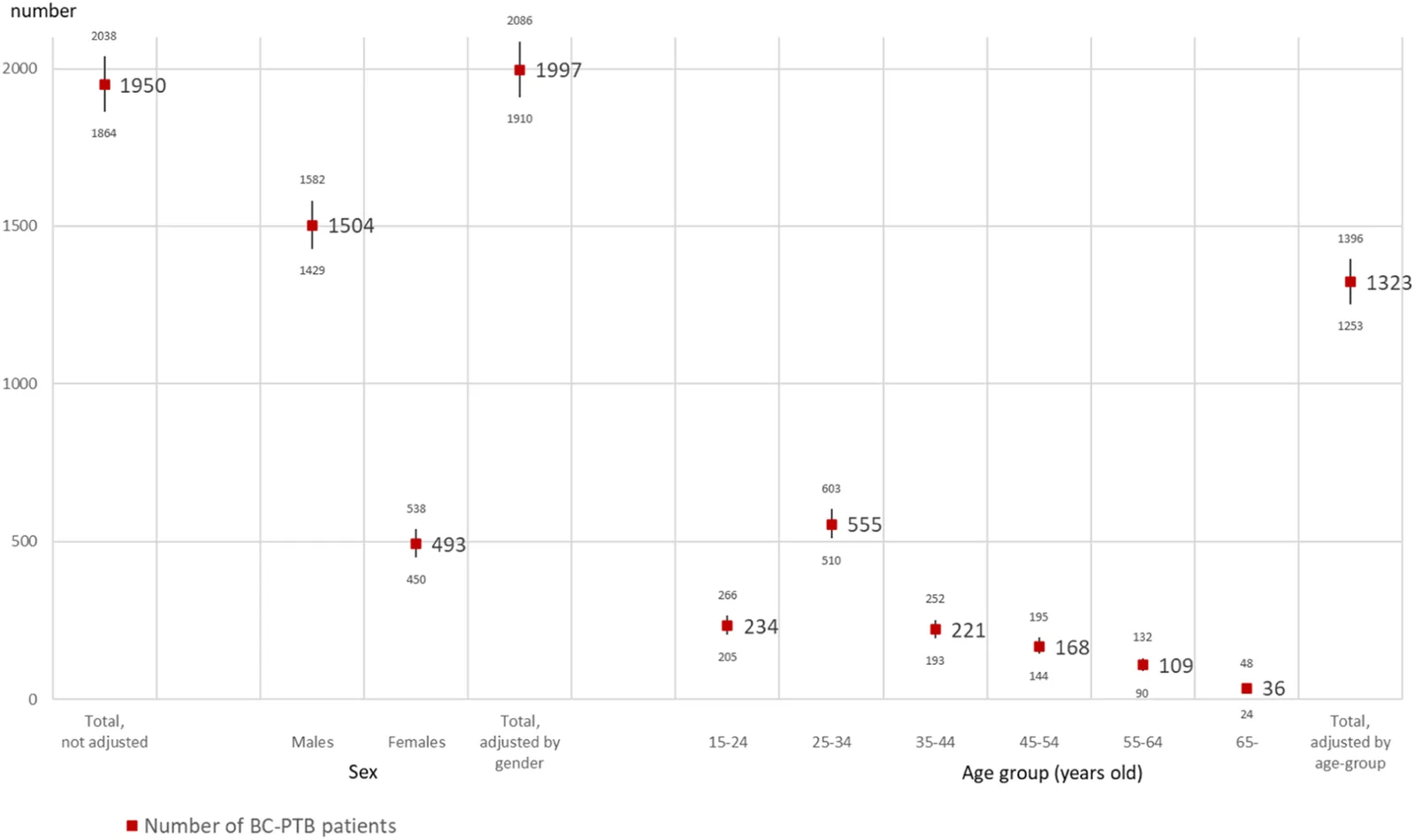
Estimated number of bacteriologically confirmed pulmonary TB (BC-PTB) cases to be identified by JPETS in the six JPETS targeted countries^a^, adjusted for sex and age group^b^. ^a^Targeted six JPETS programme countries: The Philippines, Nepal, Vietnam, Indonesia, Myanmar, and China. ^b^Sex and age group were adjusted to the proportion of foreign-born residents in Japan in 2024. Age group under 15 years was excluded. Vertical bars indicate 95% confidence intervals for the number of BC-PTB cases. JPETS: Japan Pre-Entry Tuberculosis Screening.

The estimated NNS to identify one BC-PTB case by the JPETS programme in the six targeted countries, assuming a 100% detection rate, was 227 (95% CI: 217–237) when adjusted for sex and 319 (95% CI: 302–337) when adjusted for age group (Figure 2). The NNS for identifying one BC-PTB case using the JPETS programme was significantly smaller among male applicants than among female applicants (155 males, 95% CI: 147–163, vs. 447 females, 95% CI: 409–489). The NNS was also smaller in the middle-aged and older age groups (aged ≥45 years) than in the younger age groups (Figure 2).

**Figure 2.**
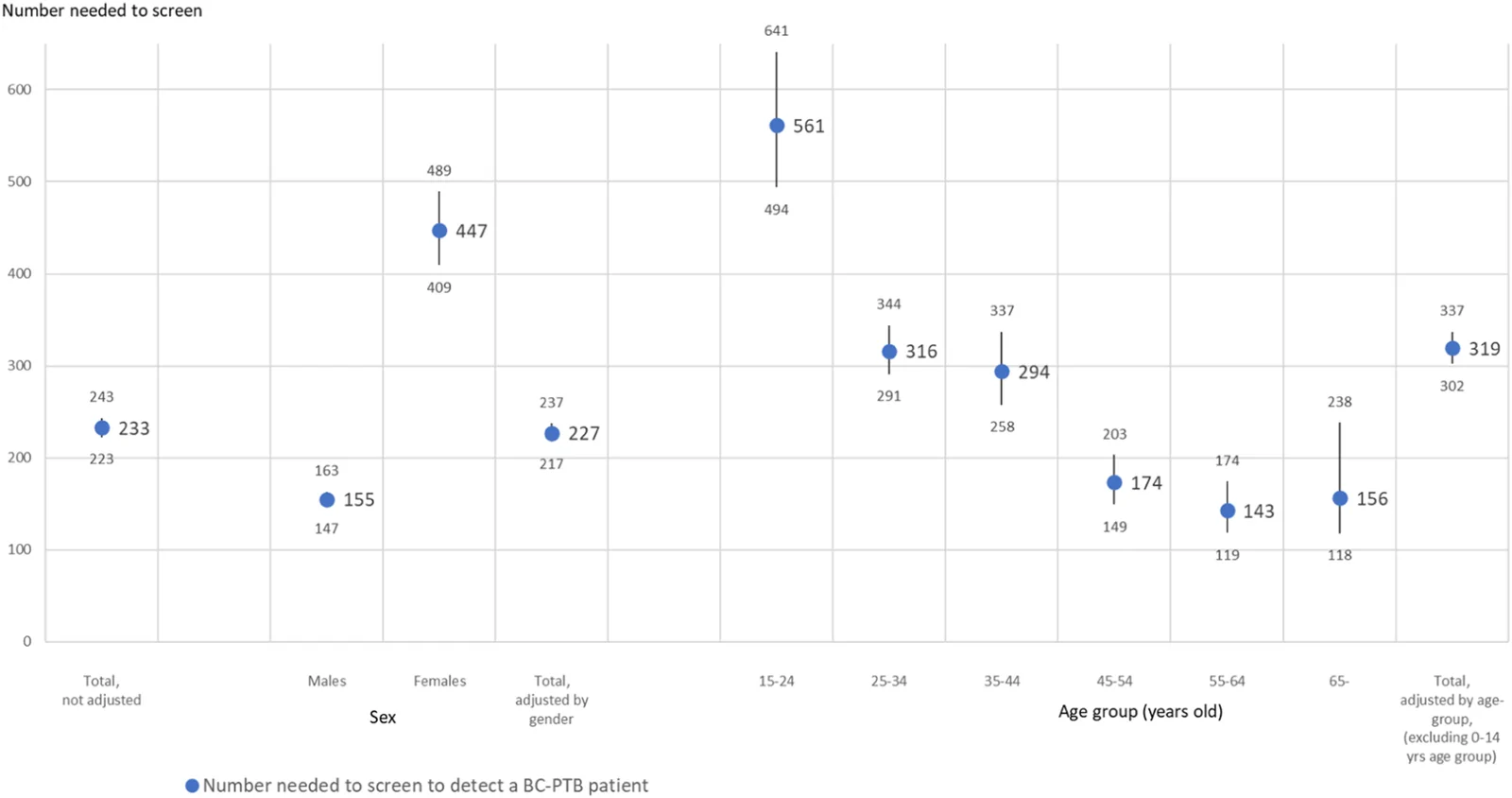
Estimated number needed to screen (NNS) to identify one bacteriologically confirmed pulmonary TB (BC-PTB) case in the targeted six JPETS countries^a^, adjusted for sex and age group^b^. ^a^Targeted six JPETS programme countries: The Philippines, Nepal, Vietnam, Indonesia, Myanmar, and China. ^b^Sex and age group were adjusted to the proportion of foreign-born residents in Japan in 2024. Age group under 15 years was excluded. Vertical bars indicate 95% confidence intervals for the number needed to screen to identify one BC-PTB case. JPETS: Japan Pre-Entry Tuberculosis Screening.

The total estimated number of BC-PTB cases that could be identified by JPETS, adjusted for age group and assuming a 100% detection rate, were relatively small in China (97 cases, 95% CI: 65–129), Nepal (108 cases, 95% CI: 60–156), and Myanmar (137 cases, 95% CI: 86–188) (Figure 3; Supplementary Data Figures S1 and S3), but large in Vietnam (193 cases, 95% CI: 106–374), Indonesia (384 cases, 95% CI: 331–407), and the Philippines (404 cases, 95% CI: 306–503) – see Supplementary Data Figures S5, S7, and S9. The estimated NNS to identify one BC-PTB case, adjusted for age group, was relatively large in China (1,030 cases, 95% CI: 772–1,536), Nepal (535 cases, 95% CI: 370–964), and Vietnam (585 cases, 95% CI: 301–1,065), respectively (Figure 4, Supplementary Data Figures S2 and S6). The NNS values were relatively small in Indonesia (176 cases, 95% CI: 132–241) and the Philippines (82 cases, 95% CI: 66–108), respectively (Supplementary Data Figures S8 and S10).

**Figure 3.**
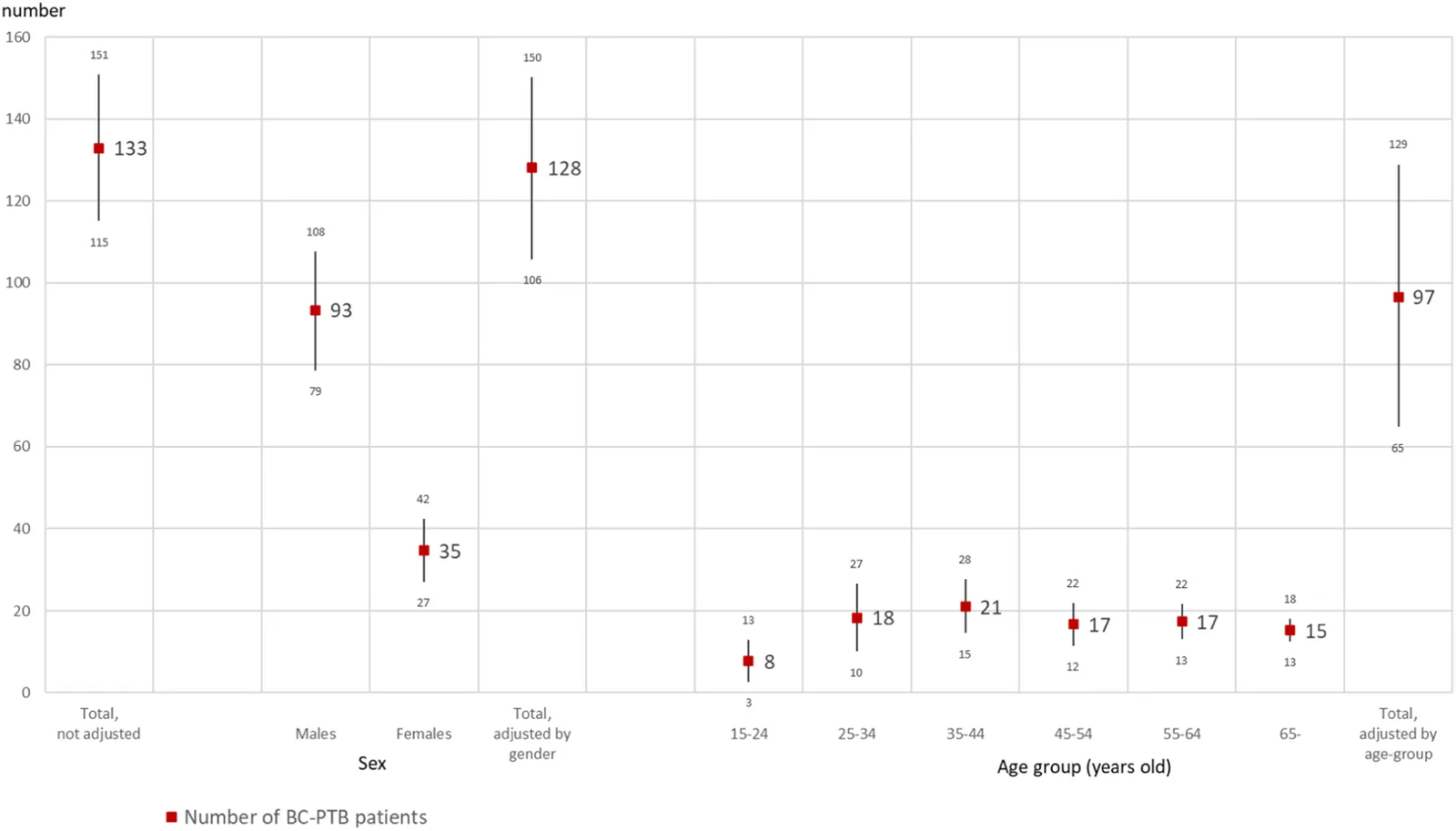
Estimated number of bacteriologically confirmed pulmonary TB (BC-PTB) cases to be identified by JPETS in China^a,b^. ^a^Sex and age group were adjusted to the proportion of foreign-born residents in Japan in 2024. Age group under 15 years was excluded. ^b^Vertical bars indicate 95% confidence intervals for the number of BC-PTB cases. JPETS: Japan Pre-Entry Tuberculosis Screening.

**Figure 4.**
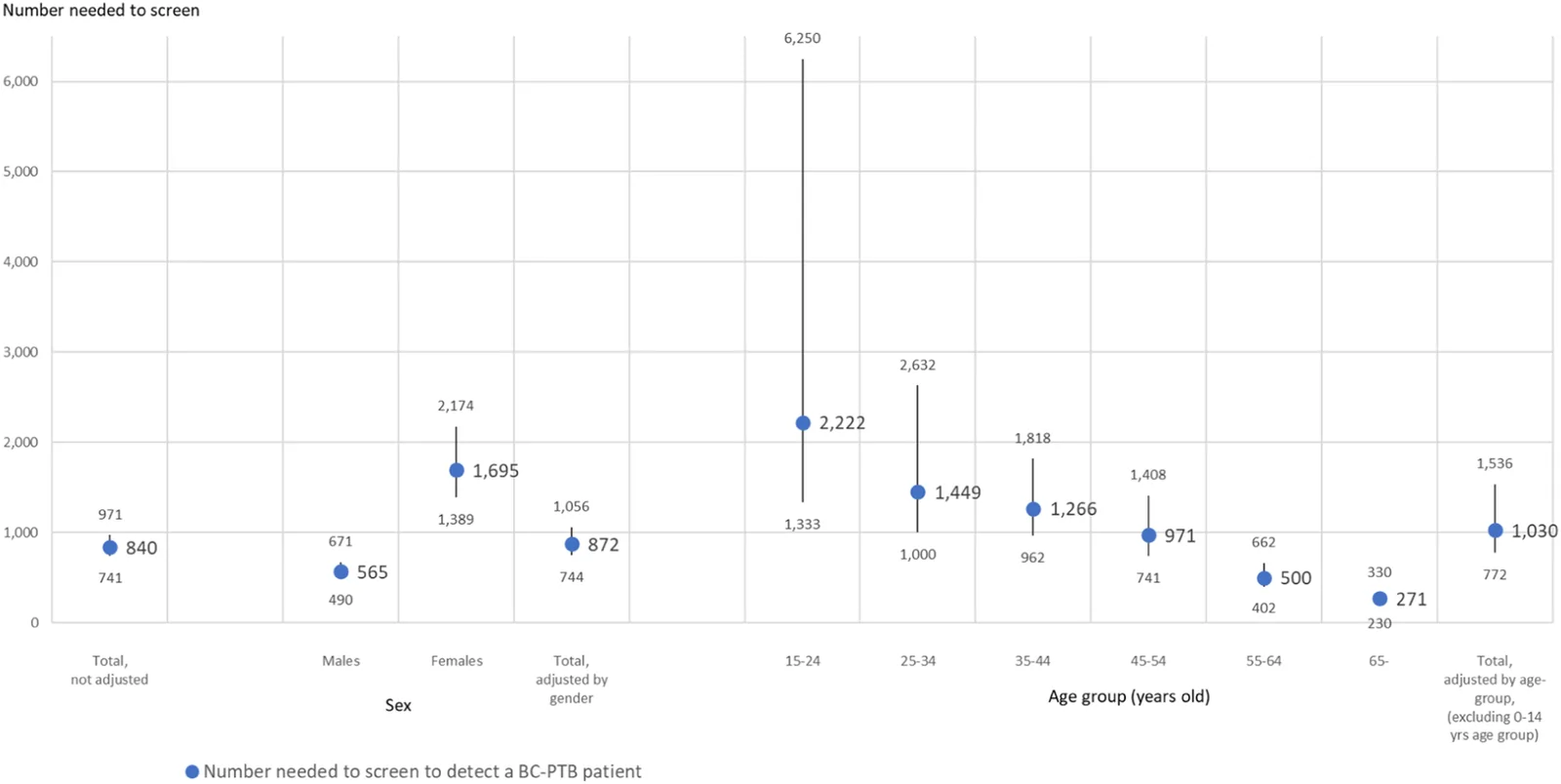
Estimated number needed to screen to identify one bacteriologically confirmed pulmonary TB (BC-PTB) case by JPETS in China^a,b^. ^a^Sex and age group were adjusted to the proportion of foreign-born residents in Japan in 2024. Age group under 15 years was excluded. ^b^Vertical bars indicate 95% confidence intervals for the number needed to screen to identify one BC-PTB case. JPETS: Japan Pre-Entry Tuberculosis Screening.

## DISCUSSION

According to the TB statistics report in Japan, 1,980 foreign-born TB cases were notified in 2024 in Japan.^2^ Among these, 1,683 cases (85.0%) were born in one of the six JPETS-targeted countries. Of the 1,554 cases with data regarding the year of entry, 1,089 (70.1%) were notified within 2 years of entry into Japan.^2^ Under the assumptions that many foreign-born TB cases notified within 2 years after entry developed TB due to infection acquired before entering Japan, and that a substantial number of TB cases have already developed TB disease, we may expect a reduction in the number of foreign-born TB cases in Japan if the JPETS is effective. For instance, the estimated number of cases with BC-PTB was 1,323 (95% CI: 1,253–1,396) when we assumed a 100% detection rate and adjusted for age groups. The number of cases with BC-PTB to be identified by JPETS overall would decline to 794 when we assume a 60% detection rate as a practical rate.

The age-group-adjusted NNS for identifying cases of BC-PTB across the six JPETS countries was estimated to be 319 (95% CI: 302–337). This estimate is much smaller than the figure reported by Trauer et al.,^13^ indicating that 1,887 applicants needed to be screened to detect one TB case by a national TB screening programme in Australia, while the TB incidence in the first 2 years after arrival in Australia was 436 per 100,000 person-years of observation.^14^ In other words, the NNS for the post-migration screening was 229, which was closer to the BC-PTB estimate in JPETS. The yield for active TB cases through pre-arrival TB screening in the EU was 1.21%,^15^ corresponding to an NNS of 82.6. Aldridge et al.^16^ previously reported a systematic review of pre-entry TB screening programmes in migrants to low-incidence countries, where the yield of culture-confirmed TB cases in countries with a prevalence of 50–149 cases per 100,000 population was 19.7 per 100,000 screened, corresponding to an NNS of 5,076, while this increased to 335.9 per 100,000 individuals screened, corresponding to an NNS of 586, in countries with a prevalence greater than 350 per 100,000 population. The median yield for active TB cases through chest X-ray screening in Europe was 0.18%,^17^ corresponding to an NNS of 556. Dasgupta et al.^18^ previously reported that the prevalence of active TB in permanent resident applicants in Canada was 0.15%, corresponding to an NNS of 667.

The NNS among those aged 45 years or older to identify a case with BC-PTB in the six JPETS countries was relatively small compared to that among those aged 44 years or younger (Figure 2). The NNS for identifying cases with BC-PTB among male applicants was lower than that for female applicants. These results indicate that JPETS can efficiently identify cases with BC-PTB among middle-aged and older male applicants.

The age-group-adjusted estimated number of cases with BC-PTB identified by the JPETS programme in China, assuming a 100% detection rate, was 97 (95% CI: 65–129) – see Figure 3. This reflects a relatively high age-group-adjusted NNS for identifying cases with BC-PTB in China: 1,030 (95% CI: 772–1,536) – see Figure 4. A relatively high NNS indicates that a large number of applicants must be screened in China to identify a single case. The low estimated prevalence of TB in China (119 per 100,000 population in 2010)^6^ is thought to be a contributing factor. As such, it is necessary to consider not only the number of TB cases who develop the disease in Japan but also the prevalence of TB in their home country when selecting the target countries for JPETS. In contrast, the age-group-adjusted estimated number of cases with BC-PTB to be identified by the JPETS programme in Indonesia and the Philippines, assuming a 100% detection rate, indicated relatively large numbers of 384 (95% CI: 331–407) and 404 (95% CI: 306–503), respectively. The NNS to identify cases with BC-PTB in the two countries was relatively low, with estimations of 176 in Indonesia and 82 in the Philippines. The JPETS programmes in both Indonesia and the Philippines require screening a smaller number of applicants to identify cases with BC-PTB than in China.

The present study has several limitations. First, the data from the NPS in the six JPETS-targeted countries may not reflect the current prevalence of TB in each country as they were collected several years ago. Therefore, it is possible that this study would have either overestimated or underestimated prevalence of TB in the six countries. Second, the NPS data provided only total prevalence of TB by sex and age group without providing data by geographic region or socio-economic status. Hence, we could not adjust estimates by these factors. Third, the NPS data did not provide information regarding the prevalence of TB in children under 15 years of age, as the surveys did not cover children. Therefore, we excluded the prevalence of TB in children from the estimation, which may have led to an underestimation of the overall number of cases with PTB identified by JPETS. The extent of this exclusion was probably limited because the number of TB cases was very small, that is, less than 5% among all foreign-born TB cases notified in Japan.^2^ Fourth, the statistics on immigrants required to obtain the certificate of eligibility did not provide data by gender and age group.^11^ Therefore, we used the data from the statistics of foreign-born residents in Japan instead.^12^ The distribution of genders and age groups in this data may differ from that of newly entering immigrants. Fifth, some people were exempt from the JPETS process.^5^ We could not obtain the number of exempt individuals; thus, the estimated number of cases with TB in this study may have been overestimated. Despite these limitations, we estimated both the number of PTB cases that JPETS would identify and the NNS required to identify a case with BC-PTB, using data from precisely planned and nationally conducted NPS in each of the JPETS-targeted countries.

## CONCLUSION

The JPETS may identify approximately 1,300 cases with BC-PTB per year, age-group-adjusted, across the six Asian countries targeted. The age-group-adjusted NNS for identifying cases with BC-PTB was estimated to be 319. The age-group-adjusted NNS in China was relatively large, with more than 1,000 applicants. When selecting countries for JPETS, considering not only the number of TB cases in Japan but also the TB prevalence in target countries may help to enhance screening effectiveness. The age-group-adjusted number of cases with BC-PTB per year was 384 in Indonesia and 404 in the Philippines, whereas the age-group-adjusted NNS for identifying cases with BC-PTB was estimated at 176 in Indonesia and 82 in the Philippines. If implemented effectively, JPETS could identify a substantial number of cases with BC-PTB across the six targeted Asian countries, although there is room for improvement in identifying cases with active TB.

## Supplementary Material
