# Supplementary material for: Projected detection of active pulmonary TB through the Japan pre-entry TB screening programme
Source: IJTLD Open. 2026 Oct 12;3(10):647–52. doi: 10.5588/ijtldopen.26.0270 (PMC13657019; doi:10.5588/ijtldopen.26.0270)
Supplement: Supplementary file 1 [file ijtldopen26-0270_supplementarydata1.pdf]

Supplementary Data

Figure S1 Estimated number of Bacteriologically-confirmed Pulmonary Tuberculosis (BC-PTB) cases to be identified by JPETS in Nepal[a,b]

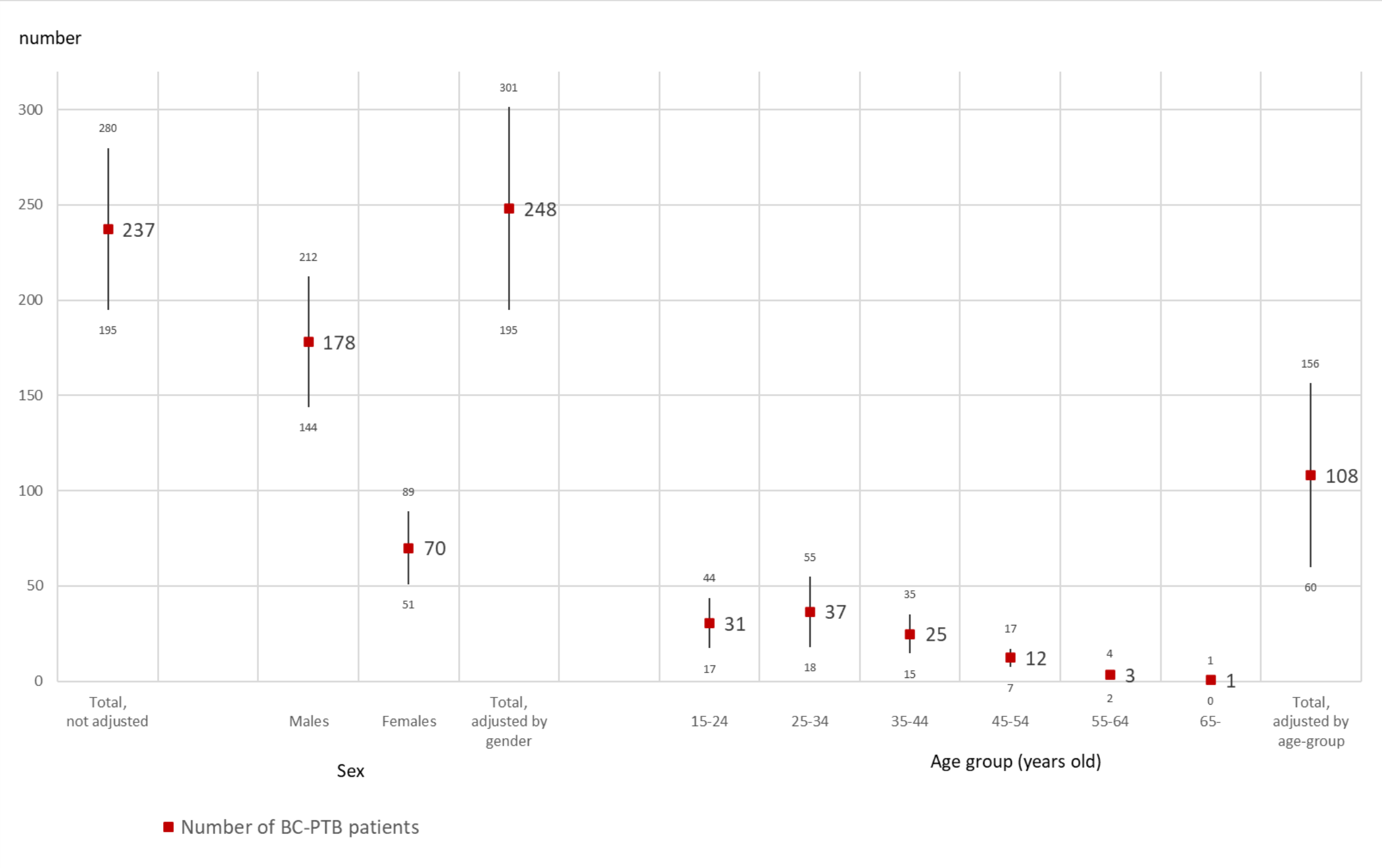

Figure S2 Estimated number needed to screen to identify one Bacteriologically-confirmed Pulmonary Tuberculosis (BC-PTB) case by JPETS in Nepal[a,c]

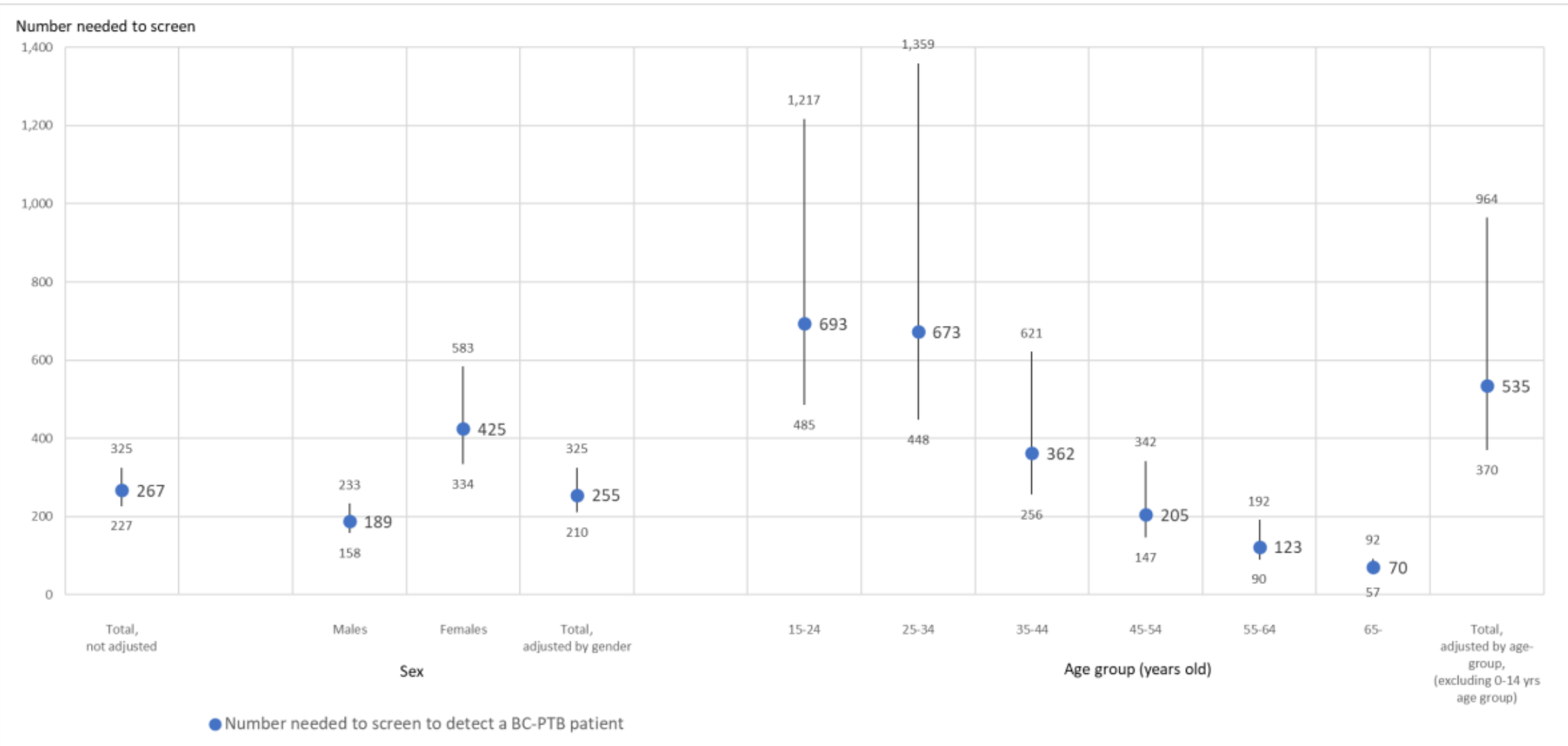

[a] Sex and age group were adjusted to the proportion of foreign-born residents in Japan in 2024. Age group under 15 years was excluded.

JPETS: Japan Pre-Entry Tuberculosis Screening; TB: Tuberculosis

[b] Vertical bars indicate 95% confidence intervals for the number of BC-PTB cases.

[c] Vertical bars indicate 95% confidence intervals for the number needed to screen to identify one BC-PTB case.

Figure S3 Estimated number of Bacteriologically-confirmed Pulmonary Tuberculosis (BC-PTB) cases to be identified by JPETS in Myanmar[a,b]

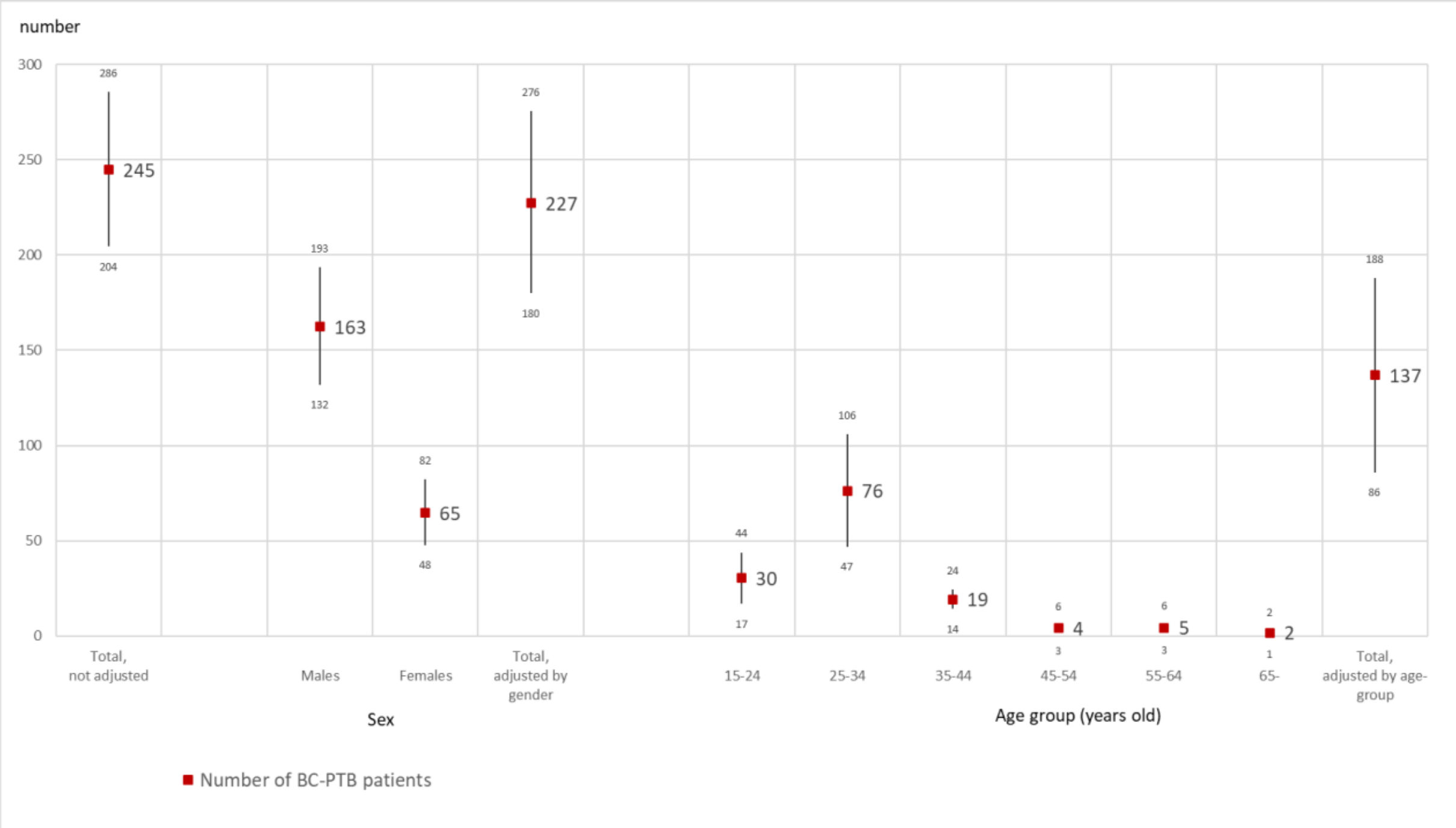

Figure S4 Estimated number needed to screen to identify one Bacteriologically-confirmed Pulmonary Tuberculosis (BC-PTB) case by JPETS in Myanmar[a,c]

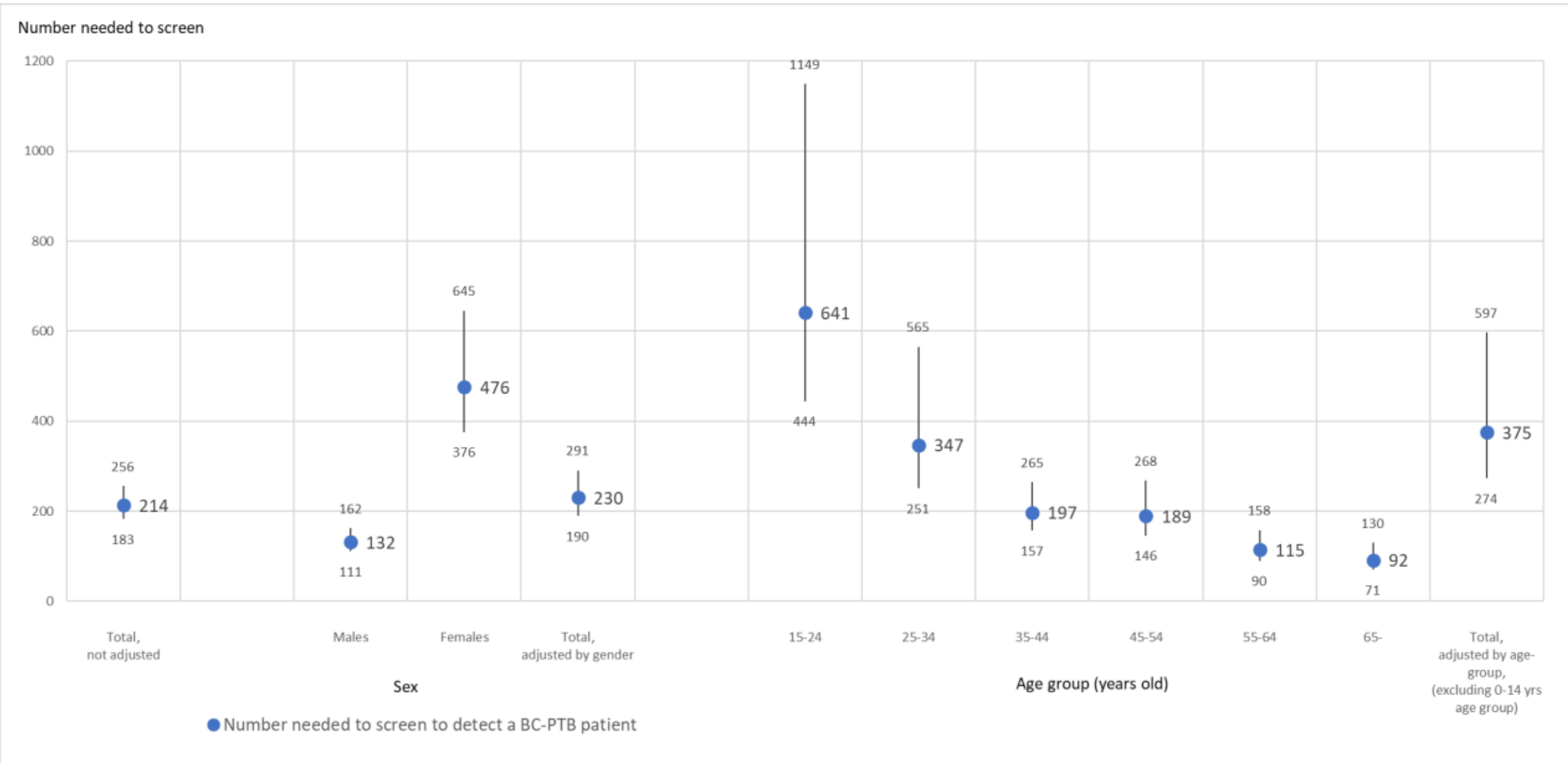

[a] Sex and age group were adjusted to the proportion of foreign-born residents in Japan in 2024. Age group under 15 years was excluded.

JPETS: Japan Pre-Entry Tuberculosis Screening; TB: Tuberculosis

[b] Vertical bars indicate 95% confidence intervals for the number of BC-PTB cases.

[c] Vertical bars indicate 95% confidence intervals for the number needed to screen to identify one BC-PTB case.

Figure S5 Estimated number of Bacteriologically-confirmed Pulmonary Tuberculosis (BC-PTB) cases to be identified by JPETS in Vietnam[a,b]

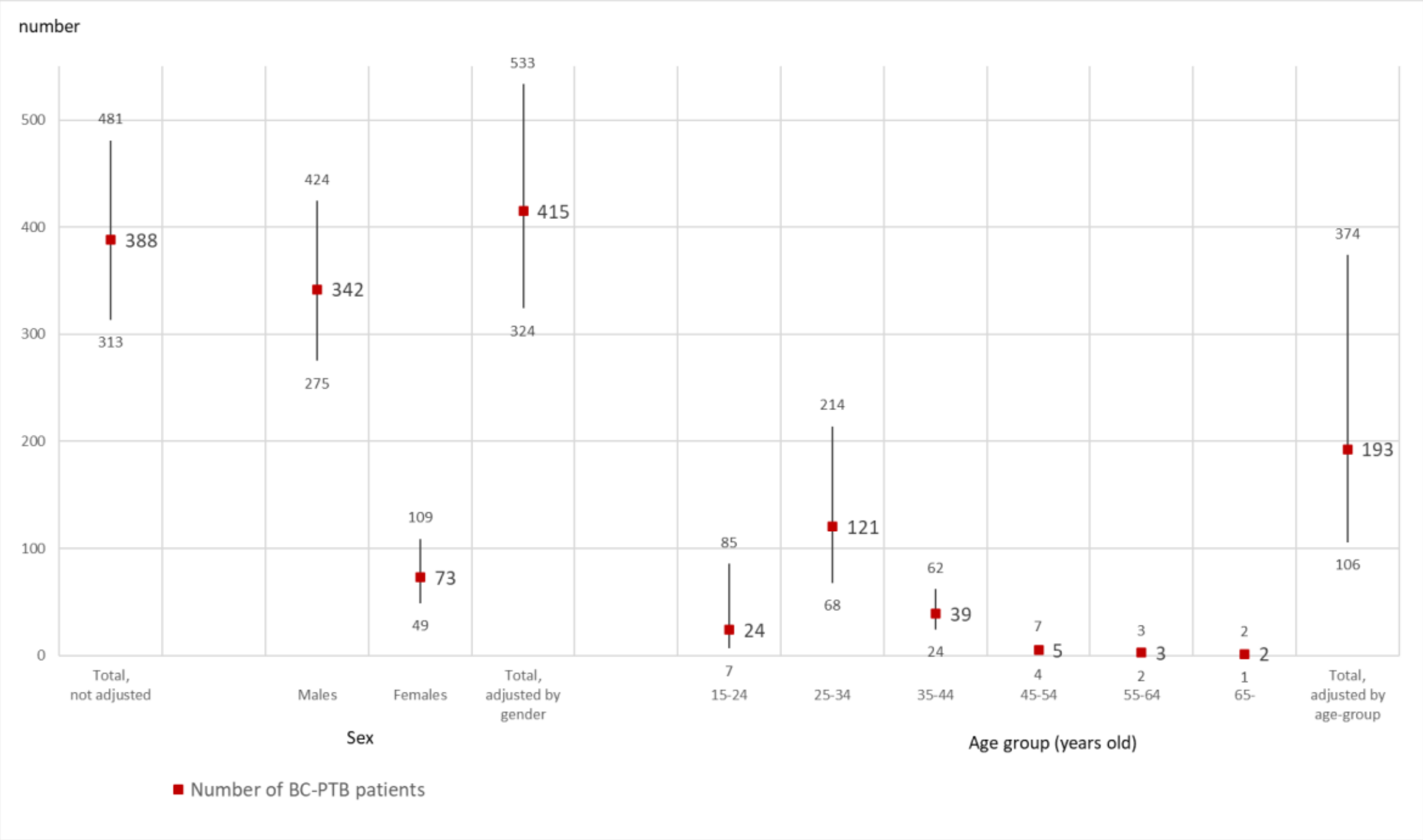

Figure S6 Estimated number needed to screen to identify one Bacteriologically-confirmed Pulmonary Tuberculosis (BC-PTB) case by JPETS in Vietnam[a,c]

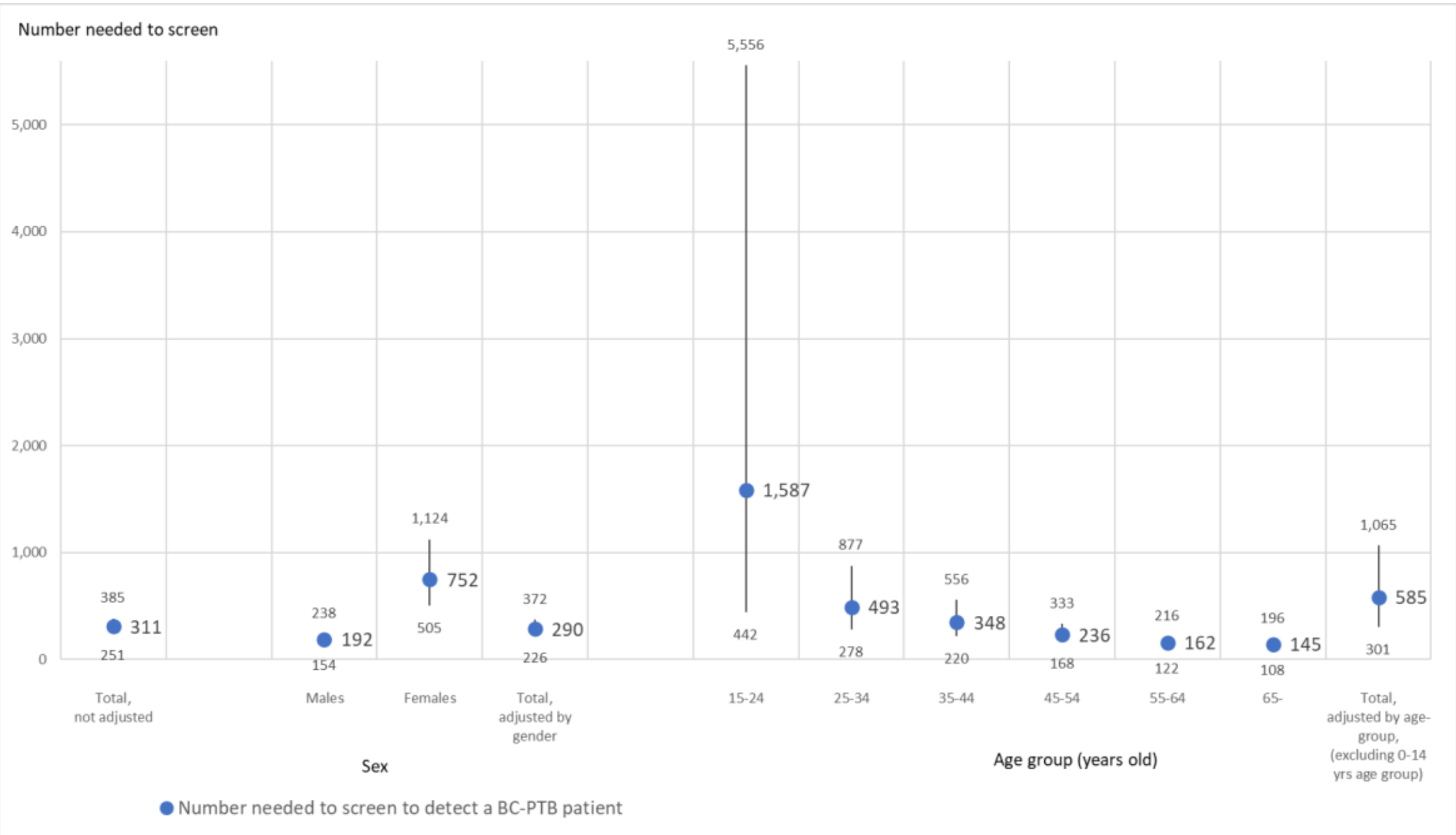

[a] Sex and age group were adjusted to the proportion of foreign-born residents in Japan in 2024. Age group under 15 years was excluded.

JPETS: Japan Pre-Entry Tuberculosis Screening; TB: Tuberculosis

[b] Vertical bars indicate 95% confidence intervals for the number of BC-PTB cases.

[c] Vertical bars indicate 95% confidence intervals for the number needed to screen to identify one BC-PTB case.

Figure S7 Estimated number of Bacteriologically-confirmed Pulmonary Tuberculosis (BC-PTB) cases to be identified by JPETS in Indonesia[a,b]

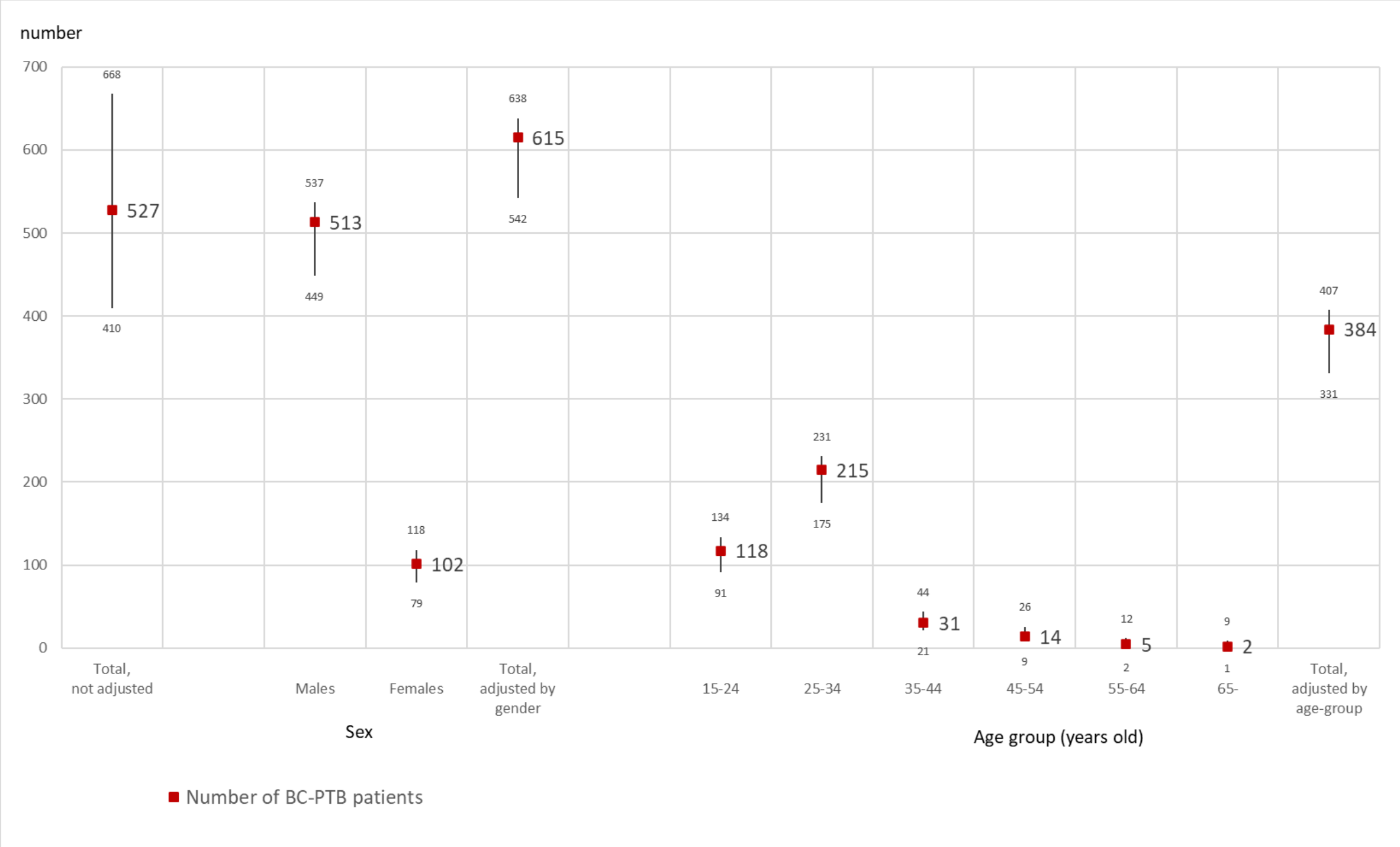

Figure S8 Estimated number needed to screen to identify one Bacteriologically-confirmed Pulmonary Tuberculosis (BC-PTB) case by JPETS in Indonesia[a,c]

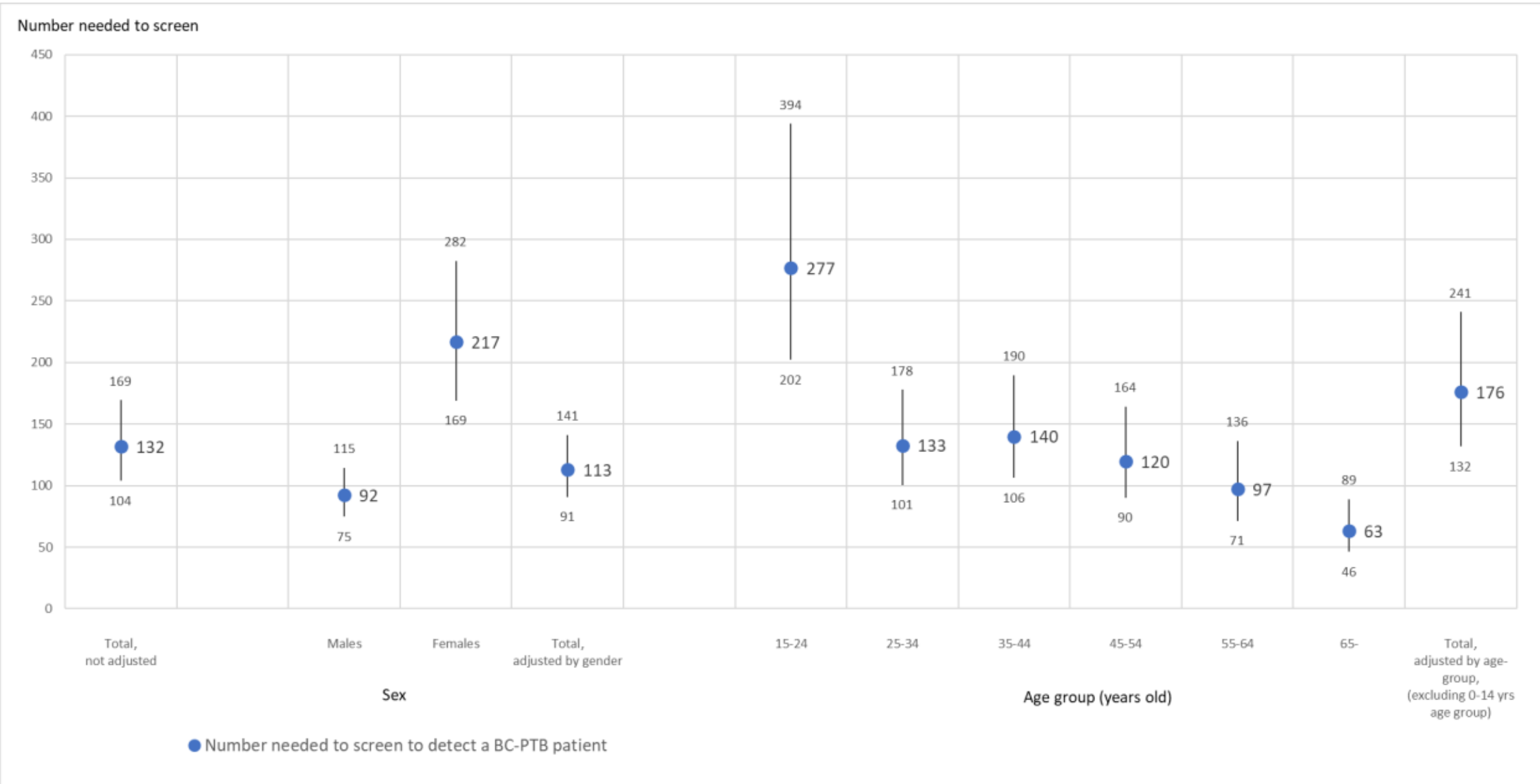

[a] Sex and age group were adjusted to the proportion of foreign-born residents in Japan in 2024. Age group under 15 years was excluded.

JPETS: Japan Pre-Entry Tuberculosis Screening; TB: Tuberculosis

[b] Vertical bars indicate 95% confidence intervals for the number of BC-PTB cases.

[c] Vertical bars indicate 95% confidence intervals for the number needed to screen to identify one BC-PTB case.

Figure S9 Estimated number of Bacteriologically-confirmed Pulmonary Tuberculosis (BC-PTB) cases to be identified by JPETS in the Philippines[a,b]

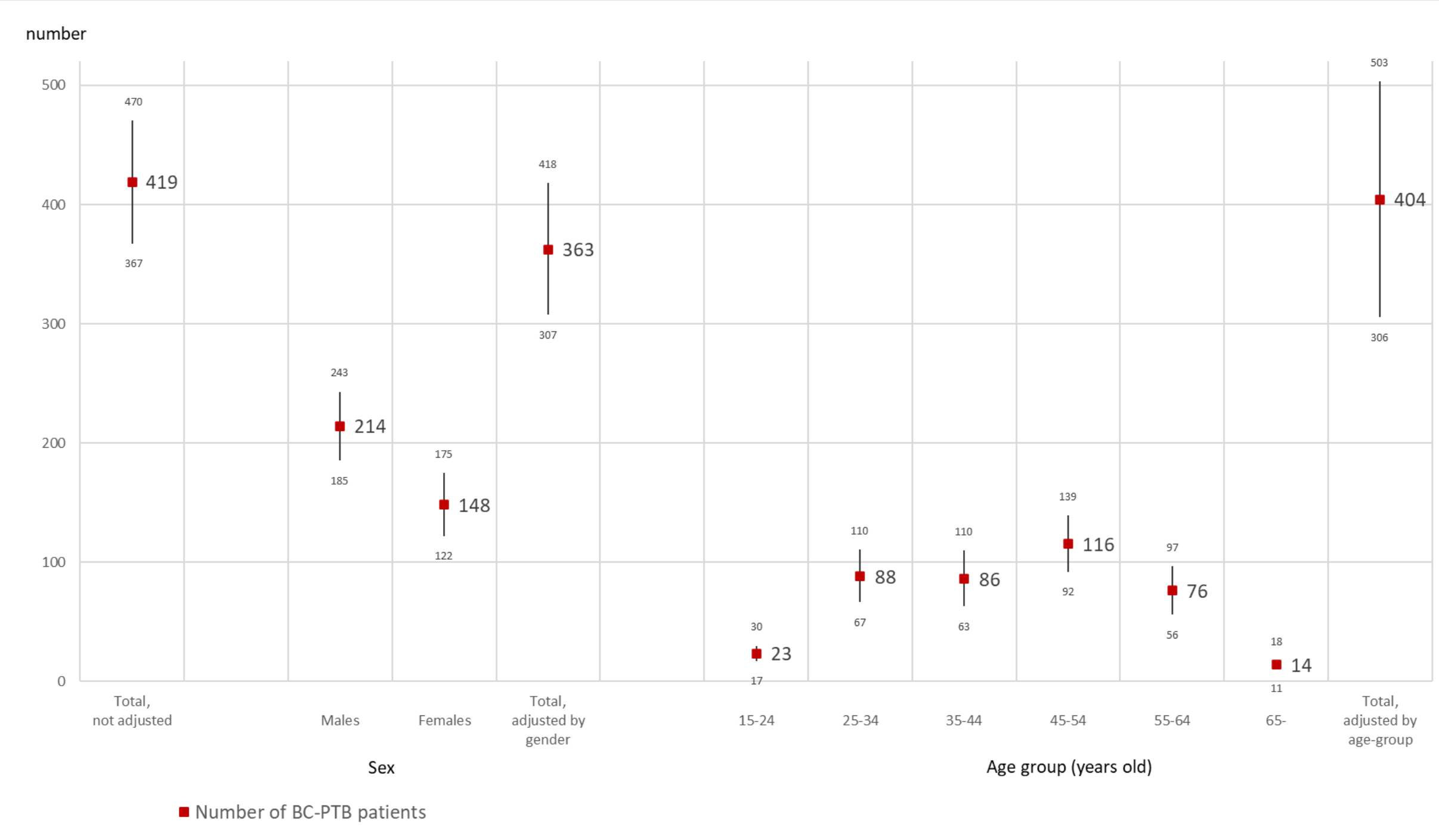

Figure S10 Estimated number needed to screen to identify one Bacteriologically-confirmed Pulmonary Tuberculosis (BC-PTB) case by JPETS in the Philippines[a,c]

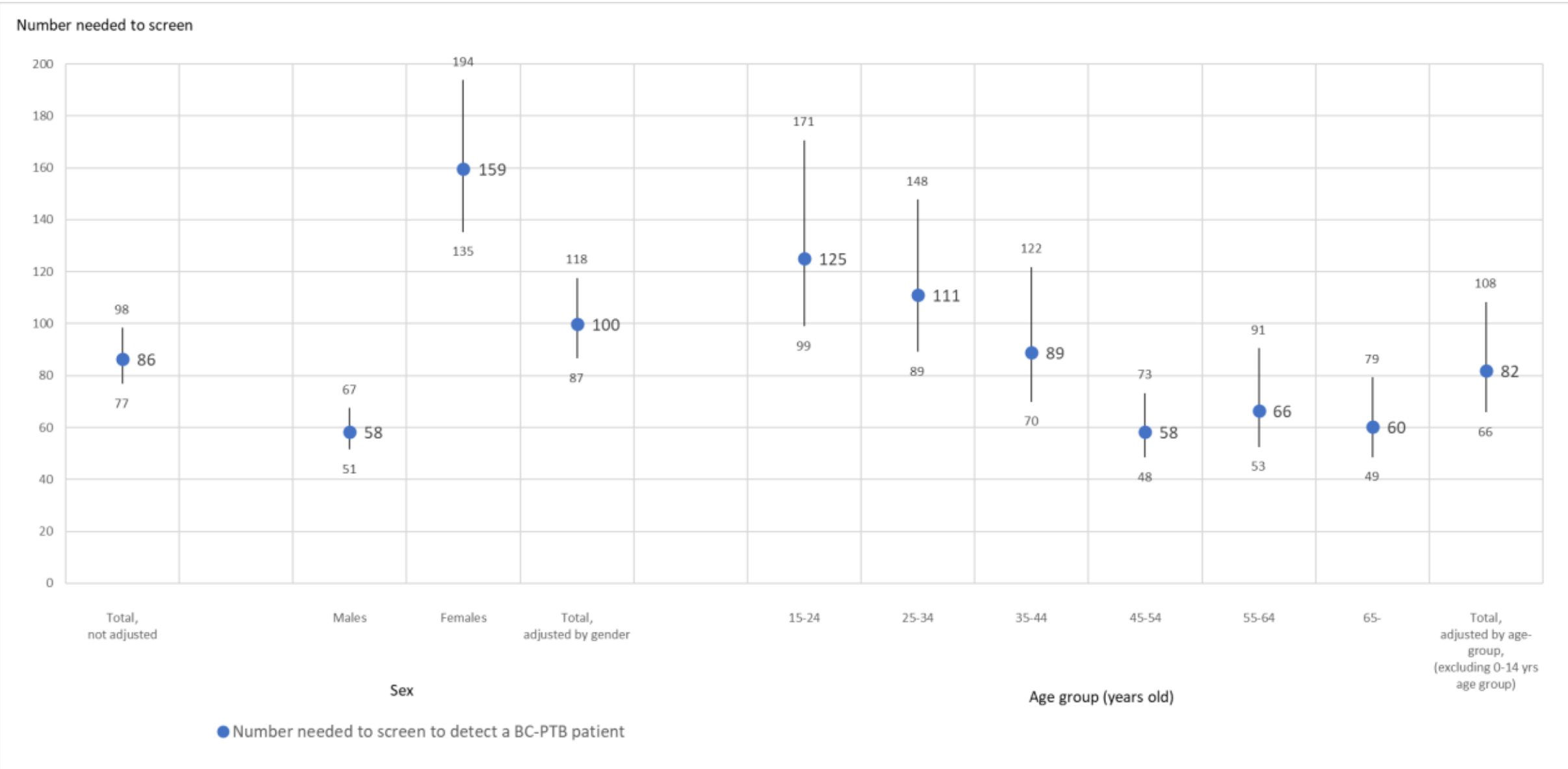

[a] Sex and age group were adjusted to the proportion of foreign-born residents in Japan in 2024. Age group under 15 years was excluded.

JPETS: Japan Pre-Entry Tuberculosis Screening; TB: Tuberculosis

[b] Vertical bars indicate 95% confidence intervals for the number of BC-PTB cases.

[c] Vertical bars indicate 95% confidence intervals for the number needed to screen to identify one BC-PTB case.
